# Arabinokinase Limits the Flux of Arabinose Into Nucleotide Sugars to Prevent Toxicity

**DOI:** 10.1002/pld3.70094

**Published:** 2025-07-22

**Authors:** Eva Ivanov Kavkova, Marion Christine Hoepflinger, Mathias Hopfinger, Wiebke Halpape, Christof Regl, Klaus Herburger, Raimund Tenhaken

**Affiliations:** ^1^ Molecular Plant Physiology, Department of Environment & Biodiversity Paris Lodron University of Salzburg Salzburg Austria; ^2^ Bioanalytical Research Labs, Department of Biosciences & Medical Biology Paris Lodron University of Salzburg Salzburg Austria; ^3^ University of Rostock Rostock Germany

**Keywords:** *Arabidopsis thaliana*, arabinogalactan protein, Arabinokinase, L‐arabinose, nucleotide sugar, recycling pathway, sugar toxicity

## Abstract

Arabinokinase (ARA1) is a key player in the recycling pathway of the major cell wall component L‐arabinose (L‐Ara). The enzyme catalyzes phosphorylation of L‐Ara to L‐arabinose‐1‐phosphate, which is then converted into UDP‐L‐arabinopyranose (UDP‐L‐Ara*p*) by UDP‐sugar pyrophosphorylase (USP) followed by conversion into UDP‐L‐arabinofuranose (UDP‐L‐Ara*f*) by UDP‐arabinopyranose mutases (UAM) before it is incorporated into cell wall polymers. While this pathway is typically nonessential for plant development, a threefold accumulation of UDP‐L‐Ara*p* can lead to toxicity. To investigate this, we generated *Arabidopsis thaliana* lines overexpressing the kinase domain of ARA1 (ARAK1‐OE) and examined their response to L‐Ara feeding. ARAK1‐OE seedlings revealed dose‐dependent root growth retardation and cell death. The presence of 3 mM L‐Ara resulted in an eightfold increase in UDP‐L‐Ara*p* levels compared with nonfeeding conditions. Interestingly, wildtype seedlings showed no visible phenotype regardless of available L‐Ara and despite the increase in UDP‐L‐Ara*p*, suggesting a critical threshold for the observed phenotype. Cell walls of ARAK1‐OE revealed a stronger attachment of arabinogalactan proteins (AGPs). Gene expression analysis from seedlings grown on 3 mM L‐Ara implied that accumulation of UDP‐L‐Ara in ARAK1‐OE triggers cell death resembling pathogen‐induced hypersensitive responses. Overall, our findings demonstrate that modest increases in UDP‐L‐Ara*p* levels can lead to significant phenotypic effects, including programmed cell death. This study highlights the role of arabinokinase in regulating L‐Ara flux into nucleotide sugars, preventing arabinose‐induced toxicity, and offers novel insights into the regulatory function of arabinokinase in cell wall biosynthesis and plant stress responses.

## 1 Introduction

Arabinose (Ara) is a common plant pentose, present in plants as the L‐isomer (L‐Ara). It is one of the primary components of plant cell walls and is found in arabinoxylan, arabinogalactan proteins (AGPs), rhamnogalacturonan I (RG‐I) side chains, and water‐soluble cytoplasmic heteroglycans (SHGs) (Fettke et al. 2005; Kotake et al. 2007; Konishi et al. 2011). Prior to being incorporated into cell wall polymers, L‐Ara must be activated into UDP‐L‐Ara (uridine diphosphate‐L‐arabinose) to serve as a substrate for glycosyltransferases. In plants, two pathways are known to activate L‐Ara. The primary pathway is the de novo synthesis, in which UDP‐xylose is epimerized to UDP‐L‐Ara. UDP‐xylose is derived from photosynthesis, starting with the oxidation of UDP‐glucose to UDP‐glucuronic acid, followed by decarboxylation. The second pathway is the recycling of free arabinose, released during growth or cell wall remodeling. Roots may also take up L‐Ara from soil, where it is released by bacterial or fungal degradation processes (Gunina and Kuzyakov 2015). In this recycling pathway, L‐Ara is phosphorylated to L‐arabinose‐1‐phosphate (L‐Ara‐1P) by arabinokinase (ARA), and subsequently converted to UDP‐L‐Ara by UDP‐sugar pyrophosphorylase (USP), which has a broad substrate specificity for various sugar‐1‐phosphates. Unlike the de novo pathway, which occurs in the Golgi apparatus, the recycling pathway takes place in the cytosol (Litterer et al. 2006; Bar‐Peled and O'Neill 2011; Geserick and Tenhaken 2013). UDP‐L‐arabinose exists in plants in two isoforms: UDP‐L‐arabinopyranose (UDP‐Ara*p*) and UDP‐L‐arabinofuranose (UDP‐Ara*f*). While the pyranose form is more stable and predominant in solution, L‐Ara is mostly incorporated into cell wall polysaccharides in the furanose form. To convert UDP‐L‐Ara*p* into UDP‐L‐Ara*f*, UDP‐L‐Ara*p* must be exported from the Golgi into the cytoplasm, where UDP‐arabinopyranose mutases (UAM) catalyze its conversion. The resulting UDP‐L‐Ara*f* is then reimported into the Golgi lumen (Rautengarten et al. 2011). The enzyme arabinokinase consists of two major domains: an N‐terminal putative glycosyltransferase and a C‐terminal sugar kinase domain. *Arabidopsis thaliana* encodes two highly similar arabinokinase isoforms, ARA1 and ARA2, which exhibit different expression patterns. Previous studies have shown that ARA1 is the more important isoform, as a knockout of ARA1 (*ara1‐2*) leads to a significant accumulation of free L‐Ara when plants are grown in soil. In contrast, an ARA2 (*ara2‐1*) knockout mutation has little impact under the same conditions (Behmüller et al. 2016).

Interestingly, even a double knockout of both ARA1 and ARA2 (*ara1‐2 ara2‐1*) does not result in obvious growth defects, as seedlings grow normally on MS‐agar plates with or without L‐Ara supplementation (Behmüller et al. 2016). These findings suggest that the arabinose recycling pathway is not essential for plant growth and development. However, the *ara1‐1* mutant, first described by Dolezal and Cobbett (1991), is hypersensitive to L‐Ara and dies within a few days on MS‐agar plates containing 5‐ to 10 mM L‐Ara. The *ara1‐1* mutant carries a single amino acid substitution (E655K) in its kinase domain, reducing the enzyme's activity to ~1% of WT levels (Dolezal and Cobbett 1991; Sherson et al. 1999; Behmüller et al. 2016). Despite its reduced enzyme activity, the *ara1‐1* mutant accumulates significantly higher levels of UDP‐L‐Ara (15‐fold increase compared with WT) when fed with L‐Ara (Behmüller et al. 2016). Furthermore, transient overexpression of arabinokinase in tobacco leaves, combined with arabinose feeding, leads to necrosis and lesions (Behmüller et al. 2016). According to these results, we stated the hypothesis that an increased activity of the kinase domain of ARA1 is responsible for L‐Ara toxicity in plants. To test this hypothesis, we overexpressed the kinase domain of ARA1 in WT *Arabidopsis* plants and investigated the resulting plants for their response to L‐Ara feeding.

## 2 Results

### 2.1 Overexpression of the Kinase Domain of ARA1 in *A. thaliana*

Previous studies indicated that elevated concentrations of UDP‐L‐Ara are responsible for L‐Ara toxicity. To test this hypothesis, we overexpressed the kinase domain of ARA1 in WT *A. thaliana* plants under the control of the 35S‐promoter (ARAK1‐OE): Two independent ARAK1‐OE lines (#7 and #25) showing different levels of overexpression were investigated. When compared with WT, ARAK1‐OE #7 revealed ~7 times and #25 ~250 times higher levels of ARAK1. Despite different transcript levels of both transgenic lines, the root phenotype is highly similar.

### 2.2 Root Growth Reduction in the Presence of L‐Ara

**FIGURE 1:**
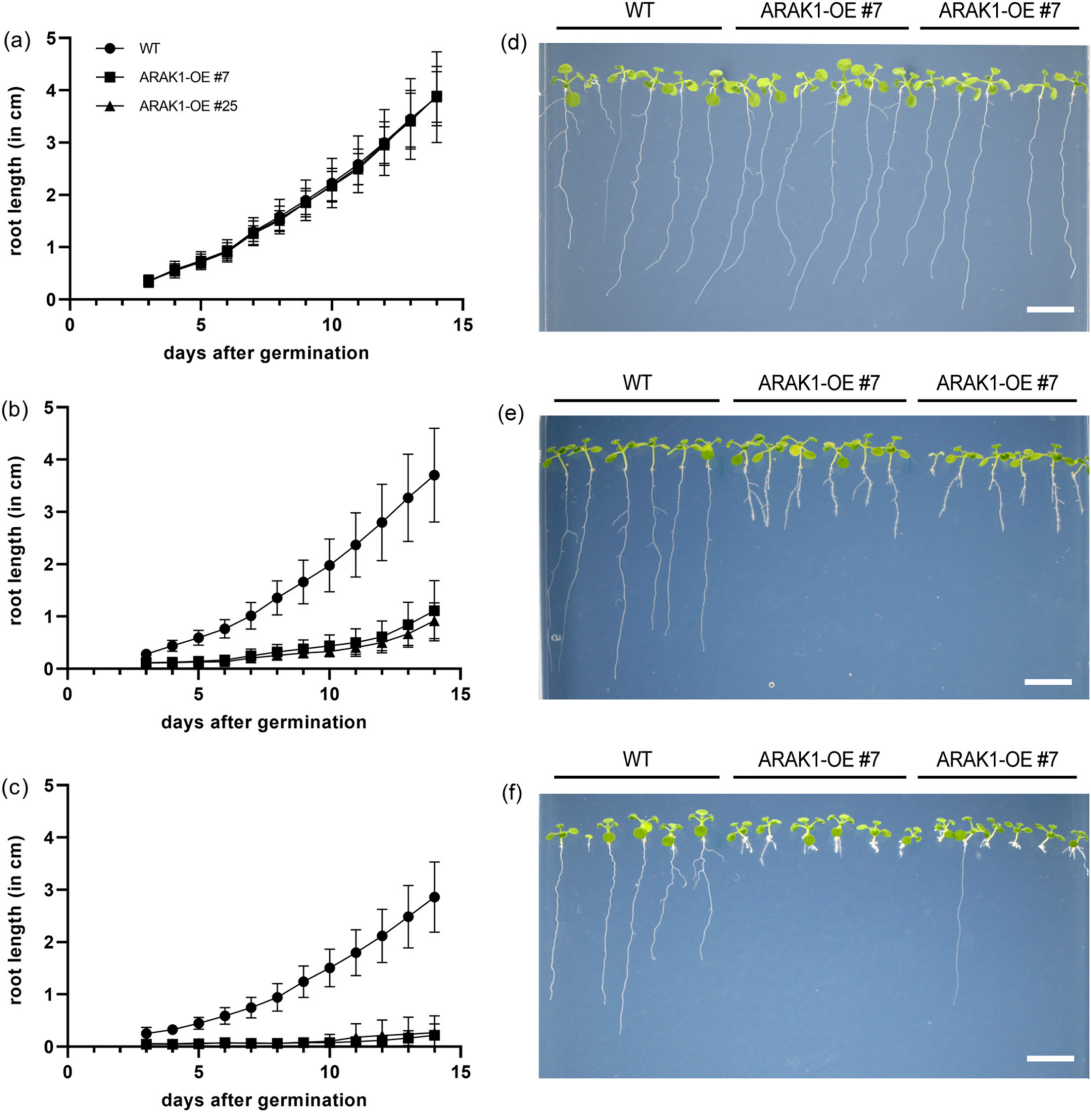
Root growth of *Arabidopsis thaliana* wild type (WT) and arabinokinase kinase domain overexpressing lines (ARAK1‐OE #7 and #25). (a—c) Root growth of WT, ARA1K‐OE #7, and #25 from Day 3 to Day 14. Seedlings were grown on 0.5× MS plates without sugar (a), with 1 mM (b) or 3 mM L‐Ara (c) and the longest root of each seedling was measured. Data represent mean ± SD (*n* = 18–24) for each line. (d–f) Photographs of 2‐week‐old seedlings of WT and ARAK1‐OE lines #7 and #25. Seedlings were grown on 0.5× MS‐agar plates without added sugar (d), with 1 mM L‐Ara (e), and 3 mM L‐Ara (f) (bar = 1 cm).

When ARAK1‐OE and WT seeds were germinated and grown on 0.5× MS‐agar plates without supplemented L‐arabinose, no phenotypic differences between them were observed (Figure 1a,d). However, when the same lines were grown on 0.5× MS‐agar plates containing the sugar L‐Ara, a strong retardation in root length was observed in the overexpressor lines and this phenotype's severity correlates with increasing L‐Ara concentrations (compare Figure 1b,c,e,f): When growing seedlings for 7 days on 1 mM L‐Ara root length of ARAK1‐OE seedlings reduced to about 20%–25% when compared with WT (1.02 ± 0.25 cm [WT], 0.25 ± 0.13 cm [ARAK1‐OE #7], 0.20 ± 0.06 cm [ARAK1‐OE #25]) and after 14 days they reduced to about 25–30% (3.70 ± 0.90 cm [WT], 1.11 ± 0.58 cm [ARAK1‐OR #7], 0.92 ± 0.34 cm [ARAK1‐OE #25]). This observed reduction in root length was even more prominent when seedlings were grown on 3 mM L‐Ara. Root length is reduced to about 9% of those of the WT after 7 days of growth (0.75 ± 0.20 cm [WT], 0.07 ± 0.02 cm [ARAK1‐OE #7], 0.07 ± 0.03 cm [ARAK1‐OE #25]) and reached about 8%–13% of the WT on day 14 (2.86 ± 0.67 cm [WT], 0.22 ± 0.22 cm [ARAK1‐OR #7], 0.27 ± 0.32 cm [ARAK1‐OE #25]). To summarize, growth of seedlings for 2 weeks in the presence of 1 mM L‐Ara led to an about 70%–80% reduction in root length of ARAK1‐OE lines while the presence of 3 mM L‐Ara caused a more than 90% reduction compared with WT. In response to L‐Ara, ARAK1‐OE plants not only exhibited the described slow but steady root growth but also developed short prominent lateral roots and showed a slight increase in root thickness (e.g., compare Figure 3d [WT] and Figure 3e [ARAK1‐OE]).

**FIGURE 2:**
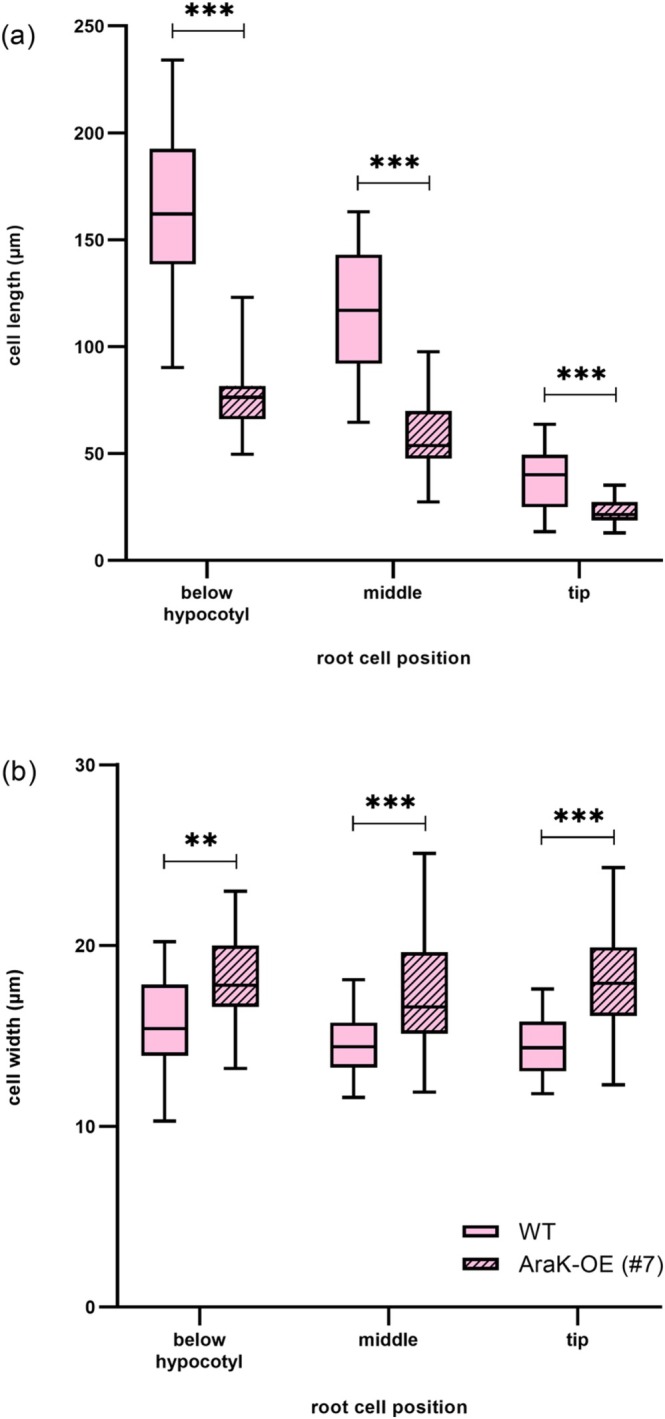
Comparison of root cell length and width in *Arabidopsis thaliana* WT and ARAK1‐OE (#7). Cell length (a) and width (b) of individual root cells of 2‐week‐old seedlings grown on 0.5× MS plates supplemented with 1 mM L‐arabinose were compared (means ± SD). For each bar, 21–34 cells from three individual seedlings were measured at different positions along the roots: directly below the hypocotyl at the beginning of the root (below hypocotyl), in the middle of the total root length (middle) and at the end of the root directly above the tip (tip). Statistical differences were calculated using unpaired *t* tests (**p < 0.01, ***p < 0.001).

To have a closer look at the root shortening, we measured the length and width of root cells at different positions along developing roots of WT and ARAK1‐OE #7 growing on 1 mM L‐Ara (Figure 2). Root cells of ARAK1‐OE #7 were significantly shorter (Figure 2a) and wider (Figure 2b) than those in WT cells. In addition, many root hairs develop in the overexpressor lines in response to growth on L‐Ara (Figure 1f). Increasing the L‐Ara concentration even further (up to 10 mM) led to tiny and often dead seedlings, mainly without a root system and typically arrested in growth at the cotyledon stage (Figure S1).

**FIGURE 3:**
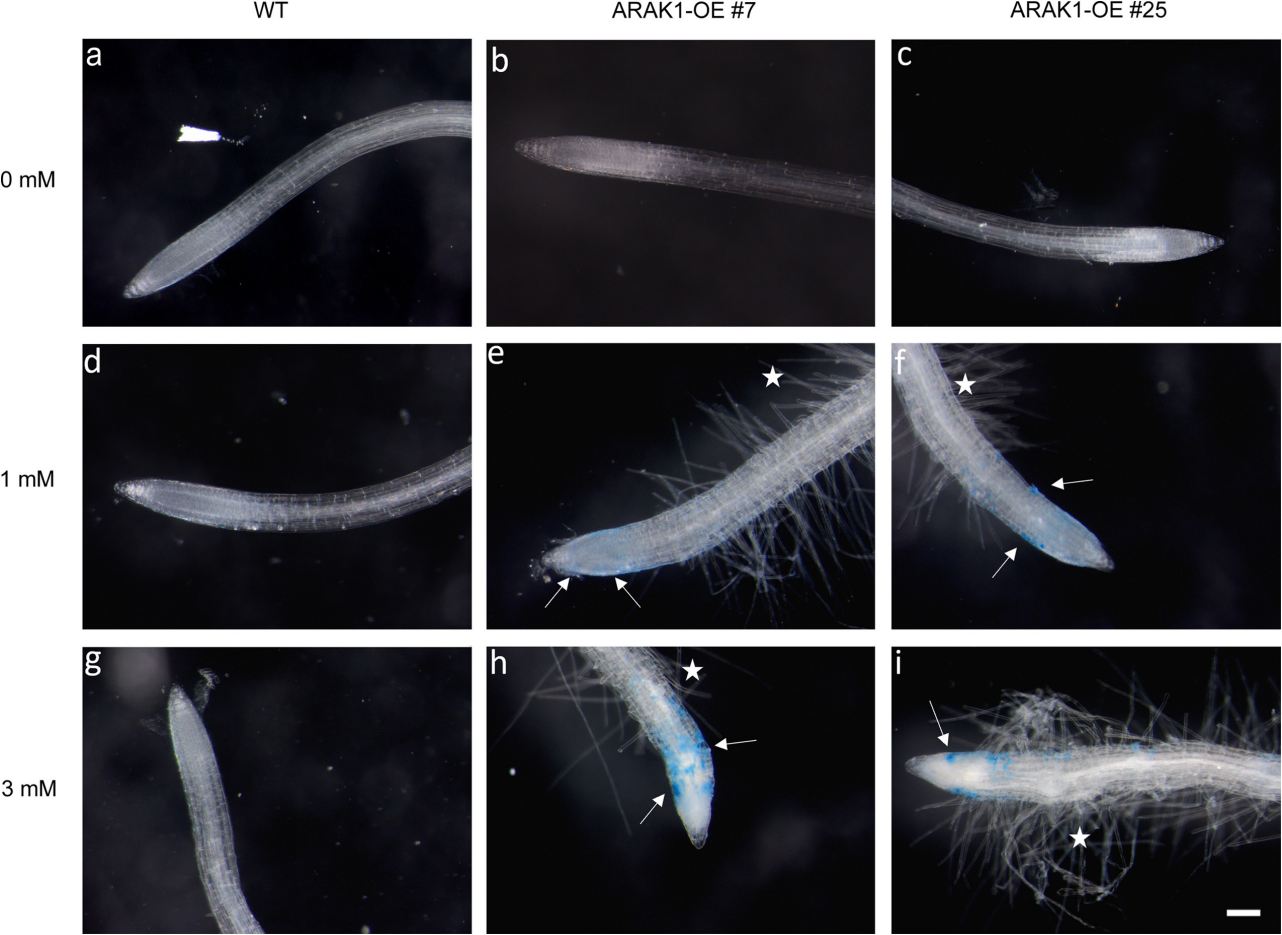
Root tips of 14 days old *Arabidopsis thaliana* seedlings stained with Evans Blue. Root tips of WT (a), ARAK1‐OE #7 (b), and #25 (c) grown without addition of L‐Ara for 14 days and stained with Evans Blue. Root tips grown on 1 mM L‐Ara: WT (d), ARAK1‐OE #7 (e), and #25 (f). Root tips grown on 3 mM L‐Ara: WT (g), ARAK1‐OE #7 (h), and #25 (i). Arrows indicate dead cells (appearing blue as stained with Evans Blue). Asterisks mark areas of sprouting root hair (bar = 100 μm).

To determine if part of the observed retardation in root growth is due to cell death, we used Evans Blue to screen for dead cells in roots in response to growth on L‐Ara. After growing for 14 days on 0.5× MS‐agar plates containing either no, 1 mM, or 3 mM L‐Ara, WT and ARAK1‐OE seedlings were carefully picked from the plate and stained with Evans Blue to visualize dead cells. No dead cells were visible in WT seedlings independent of L‐Ara concentrations (Figure 3a,d,g as well as Figure S2a,d,g), and both ARAK1‐OE lines also showed no dead cells when growing without L‐Ara supplemented. When L‐Ara was available, both ARAK1‐OE lines showed dying cells: Growth on 1 mM or 3 mM L‐Ara led to thicker roots, and stained dead cells were clearly visible at the root tip area in the elongation zone above the calyptra (stained blue; see Figure 3e,f,h,i). Beyond a concentration of 5 mM L‐Ara, no further increase in the observed phenotype was noticeable (Figure S2).

Summarizing, overexpression of ARAK1 in *A. thaliana* leads to a dramatic reduction in root growth in response to L‐Ara feeding, with root length reduced by over 90% at 3 mM L‐Ara and accompanied by shorter, thicker root cells as well as increased cell death. These findings confirm that elevated L‐Ara levels severely impair root growth in ARAK1‐OE lines.

### 2.3 UDP‐Sugar Analyses

**FIGURE 4:**
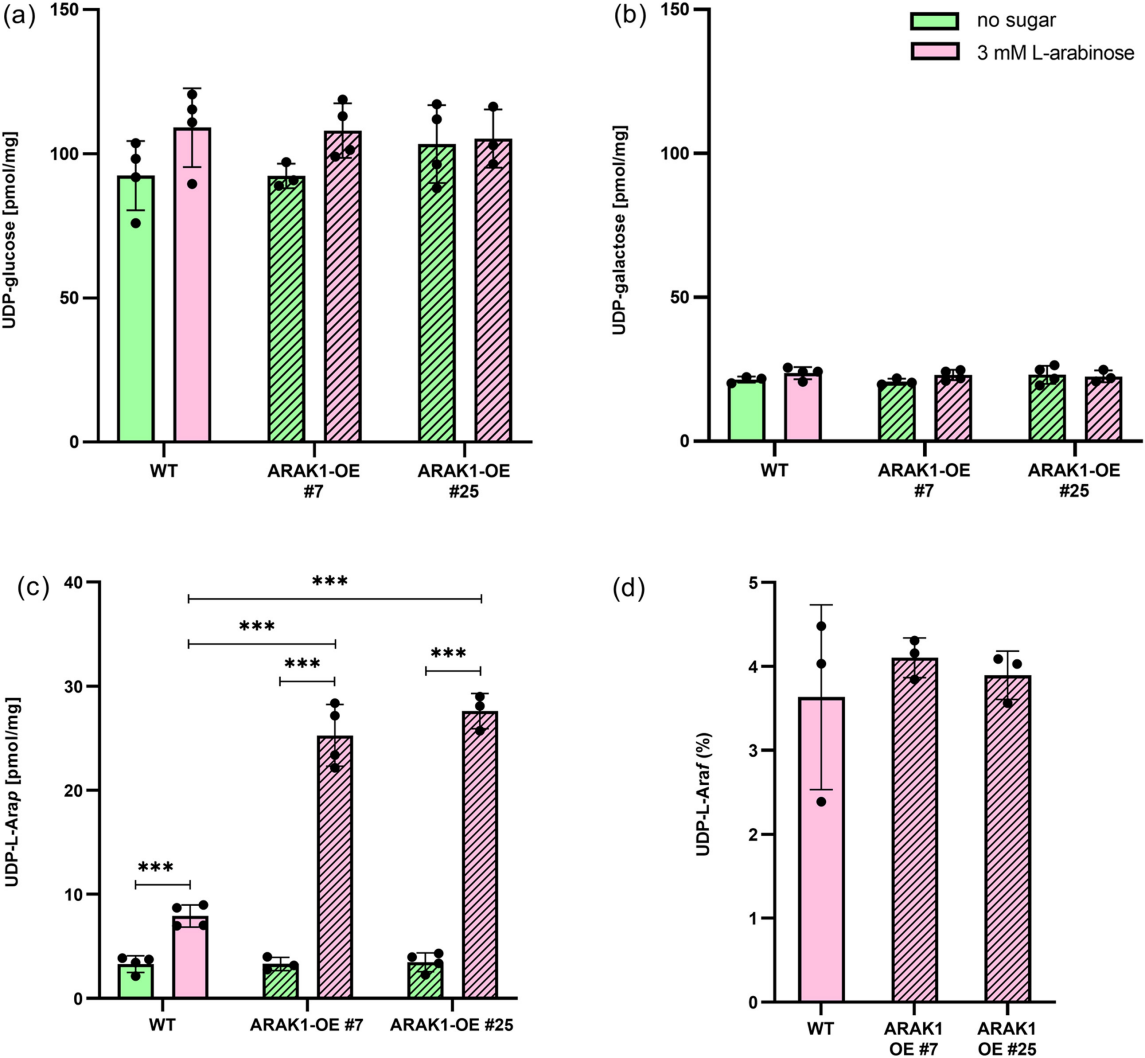
Results of nucleotide sugar analysis in seedlings grown on plates with L‐Ara. The nucleotide sugars UDP‐glucose (a), UDP‐galactose (b), UDP‐L‐arabinopyranose (UDP‐L‐Ara*p*, c), and UDP‐L‐arabinofuranose (UDP‐L‐Ara*f*, d) were measured in *Arabidopsis thaliana* wild type (WT) and two independent lines overexpressing the kinase domain of arabinokinase (ARAK1‐OE #7 and #25) grown for 14 days on 0.5× MS‐agar plates containing 3 mM L‐Ara using UHPLC‐MS (*n* = 3–4 replicates per line). Statistical differences were calculated using unpaired *t* tests and are indicated (***p < 0.001). Available amounts of UDP‐L‐Ara*f* (d) were calculated relative to the whole available pool (UDP‐L‐Ara*p* + UDP‐L‐ARA*f*) and are given in %.

To analyze potential changes in the levels of UDP‐sugars of WT and ARAK1‐OE lines growing on L‐Ara, we measured UDP‐sugars from roots of 14 days old seedlings. Independent of the presence of L‐Ara, the main UDP‐sugars in *Arabidopsis* (UDP‐glucose, UDP‐galactose) remained unchanged (Figure 4a,b), suggesting that the available UTP is sufficient to convert all available L‐Ara‐1P. On the other hand, without L‐Ara feeding, the concentration of UDP‐L‐Ara*p* was low and did not differ between WT and overexpressor lines (Figure 4c, green bars). When L‐Ara (3 mM) was available, WT seedlings significantly increased their UDP‐L‐Ara*p* pool (2.4‐fold; Figure 4c). Interestingly, the overexpressor lines accumulated much higher UDP‐L‐Ara*p* levels: an about 7.8‐fold increase compared with nonfeeding conditions was measured. As most UDP‐L‐Ara in cell wall polymers is present in the furanose form, we looked at the furanose levels as well. Despite the changes in UDP‐L‐Ara*p* levels in response to L‐Ara feeding, the relative amounts of UDP‐L‐Ara*f* were the same in all lines and reached approximately 3.8% of the total UDP‐L‐Ara pool (Figure 4d). Thus, the ratio between UDP‐L‐Ara*p* and UDP‐L‐Ara*f* remained constant and was independent of the UDP‐L‐Ara*p* pool size.

We also had a look at the cell wall sugar composition of WT and ARAK1‐OE lines (Figure S4): Similar results were observed in all cell wall sugars when WT and ARAK1‐OE #7 and #25 were compared. These results provide no evidence for a major increase of L‐Ara‐based polymers.

Overall, UDP‐sugar profiling revealed that major UDP‐sugars remained unchanged, while ARAK1‐OE lines accumulated significantly higher levels of UDP‐L‐Ara*p* in response to L‐Ara feeding, without altering the ratio of its furanose form or cell wall sugar composition. These findings suggest that overexpression of ARAK1 elevates UDP‐L‐Ara*p* levels without increasing L‐Ara incorporation into cell wall polymers.

### 2.4 Cell Wall Analyses

**FIGURE 5:**
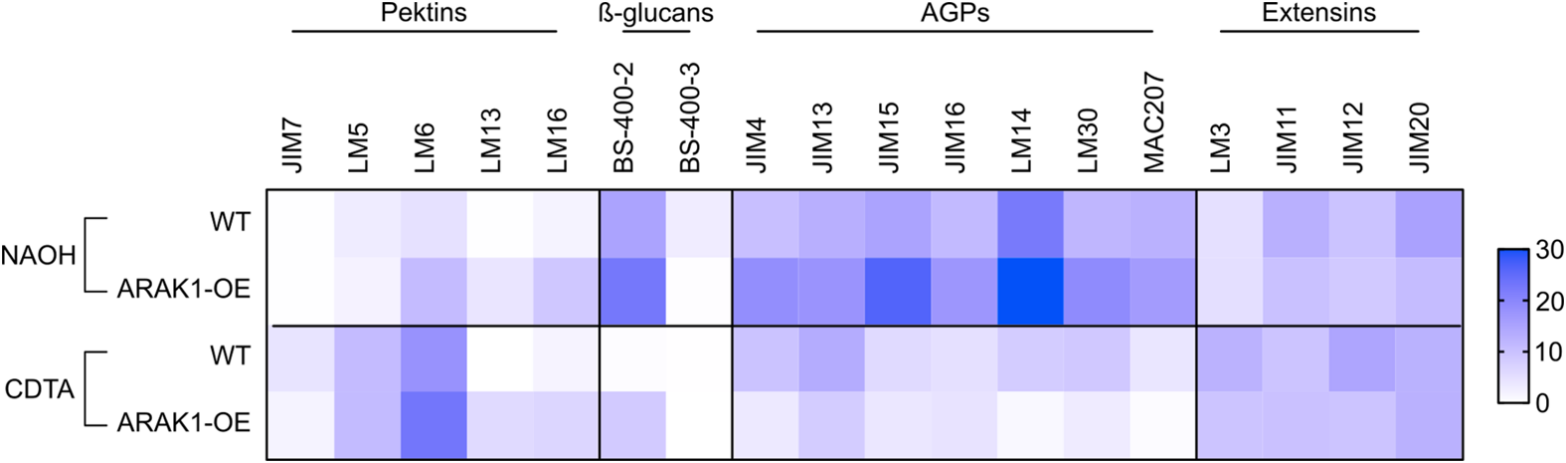
Comprehensive microarray polymer profiling (CoMPP) from *Arabidopsis thaliana* wild type and ARAK1‐OE seedlings grown on 3 mM L‐Ara. Analysis of cell wall composition by carbohydrate profiling is shown. Alcohol‐insoluble residue (AIR) samples were isolated from roots of wild type (WT) and ARAK1‐OE mutants growing for 14 days on 0.5× MS‐agar plates supplemented with 3 mM L‐Ara. Cell wall components were extracted from AIR samples with diaminocyclo‐hexane‐tetra‐acetic acid (CDTA) and NaOH. Blue colors indicate increased intensity of respective antibody binding according to the legend.

It is assumed that ARAK1‐OE plants contain higher levels of L‐Ara containing polymers in their cell walls, as the excess UDP‐L‐Ara*p* is likely incorporated into the cell wall over time, even though the levels of UDP‐L‐Ara*f* do not differ between OE and WT plants (Figure 4d). Comprehensive microarray polymer profiling (CoMPP) was chosen to examine the sugar polymers of the cell wall in WT and ARAK1‐OE plants grown on 3 mM L‐Ara for 14 days. Two fractions, one extracted with CDTA for easier extractable cell wall components like pectins and another fraction extracted with NaOH for more tightly bound components like hemicelluloses, were spotted on a membrane to perform the antibody based CoMPP analysis (Table S1). Analysis of the spot signals from CoMPP revealed higher intensities for L‐Ara containing pectins (detected with LM6, LM13, and LM16) in ARAK1‐OE compared with wildtype in both the pectic (CDTA) as well as the hemicellulose (NaOH) fraction. Galactose containing pectin chains showed nearly the same signal intensity in OE and WT in both fractions (Figure 5). Unexpectedly, in the ARAK1‐OE, arabinogalactan proteins (AGPs) shifted from loose (pectin fraction) to stronger binding (hemicellulose fraction) with the cell wall (Figure 5). Moreover, signals for various AGPs were lower in the pectin sample and higher in the hemicellulose sample of overexpressor plants when compared with WT with an average fold‐change of 1.5 (compare Table S1). Callose detected by BS‐400‐2 was more present in the NaOH fraction in the OE compared with WT, while the extensins (LM13, JIM11, JIM12, JIM20) stayed nearly the same between OE and WT in both fractions (Figure 5).

In summary, in ARAK1‐OE plants grown on L‐Ara, CoMPP analysis revealed increased levels of L‐Ara‐containing pectins and a notable shift of AGPs to more tightly bound cell wall fractions, along with elevated callose levels.

### 2.5 ARAK1‐OE Transcriptomics and Phenomics

**FIGURE 6:**
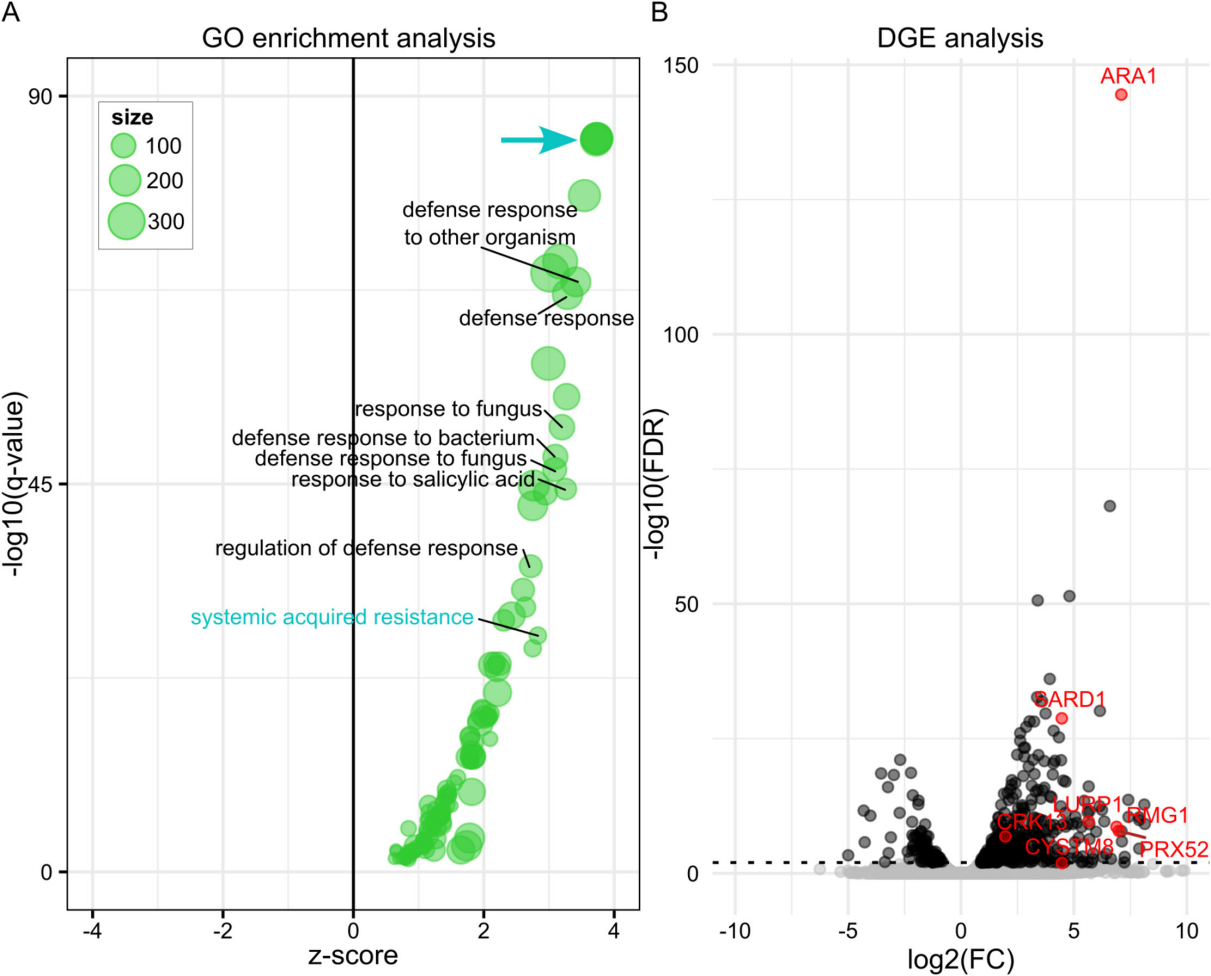
Gene Ontology (GO) enrichment and differential gene expression (DEG) analysis of ARAK1‐OE lines in response to L‐Ara. GO enrichment analysis of upregulated genes in ARAK1‐OE in response to 3 mM L‐Ara feeding. (a) Each bubble represents a significantly enriched GO term and bubble size represents the number of represented genes. The *z*‐score (*x*‐axis) refers to the ratio of up‐ and downregulated genes annotated for each GO term. Significances of enrichment analysis for each GO term is depicted on the *y*‐axis by −log10 of *q* value. Thus, the most significant terms are shown in the upper part of the plot. Notable terms are highlighted with their description. The turquoise arrow refers to four different terms (“response to external biotic stimulus,” “response to other organism,” “response to biotic stimulus,” “biological process involved in interspecies interaction between organisms”). (b) Volcano plot of DEGs. Dots represent individual genes: Significant differentially expressed genes are shown in back, not significant in gray. Red dots represent noteworthy genes of enriched terms.

**FIGURE 7:**
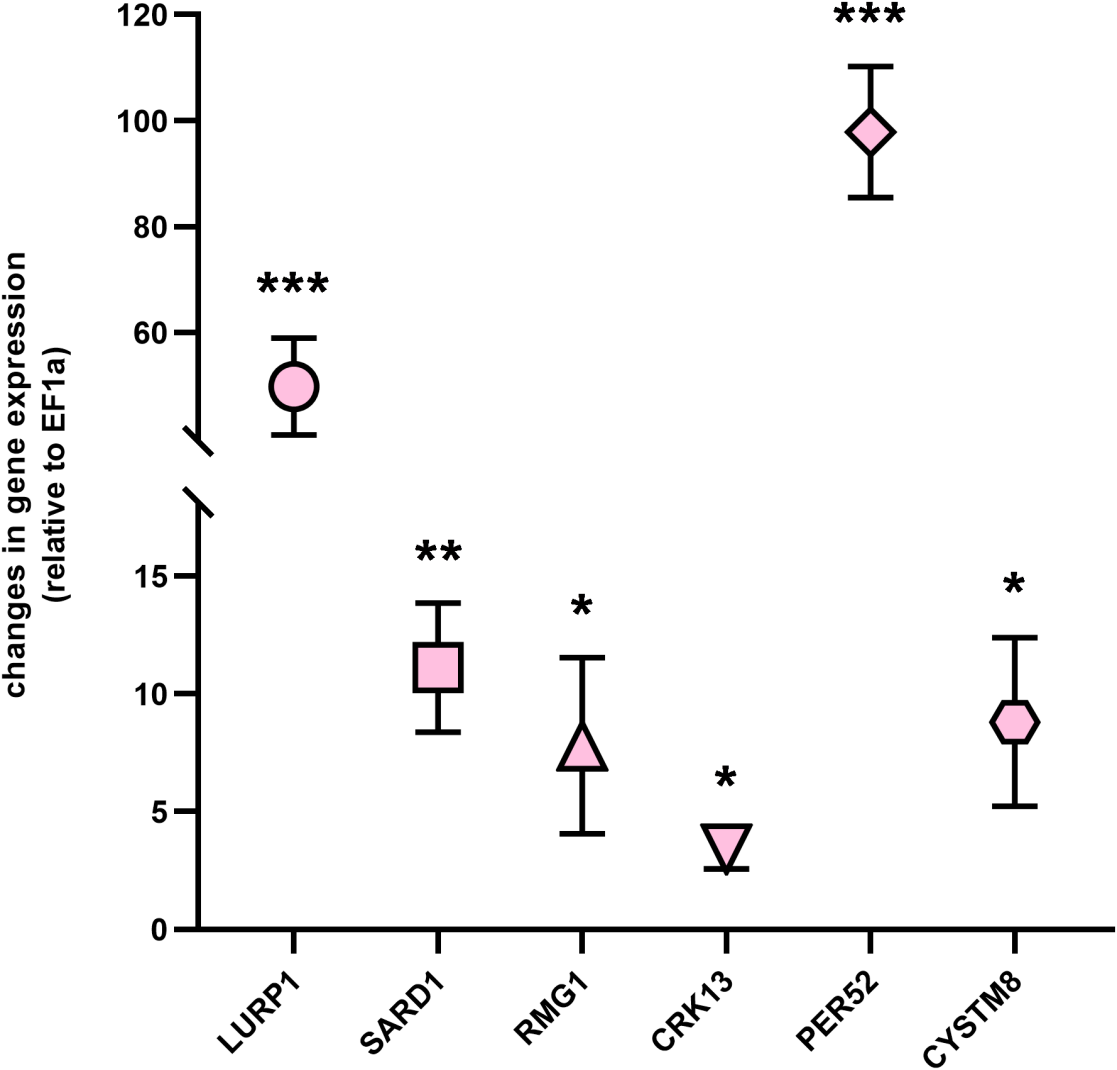
Changes in gene expression of different genes related to L‐Ara‐toxicity in *Arabidopsis thaliana* . WT and ARAK1‐OE #7 seedlings were grown on 0.5× MS‐agar plates supplemented with 3 mM L‐arabinose for 14 days. Expression levels of LURP1, SARD1, RMG1, CRK13, PER52, and CYSTM8 were measured and calculated relative to expression of EF1a (housekeeping gene). Gene expression levels of WT seedlings were set to 1 and fold changes in ARAK1‐OE #7 seedlings compared with WT are shown (means ± SD, *n* = 3). Significant differences between mean values of WT and ARAK1‐OE #7 seedlings are highlighted (unpaired *t* test, asterisks (*, **, ***) indicate difference at a level of *p* < 0.05, *p* < 0.01, *p* < 0.001).

Gene expression analysis of RNA sequencing data resulted in 422 significantly up‐ (FDR ≤ 0.01 and log2FC > 0) and 93 significantly downregulated (FDR ≤ 0.01 and log2FC < 0) genes in response to L‐Ara feeding (Figure 6b, Table S2). Gene ontology (GO) functional enrichment analyses were calculated independently for three subcategories: “biological process” (BP), “cellular component” (CC), and “molecular function” (MF). Of all upregulated genes, 135 BP terms enriched in different defense response terms and representative ones are labeled in Figure 6a. The four BP terms with the highest enrichment (turquoise arrow in Figure 6a) are clustered together and correspond to “response to external biotic stimulus,” “response to other organism,” “response to biotic stimulus,” and “biological process involved in interspecies interaction between organisms.” The highest ranked enrichments of Figure 6a correspond to general terms with more than 500 annotated members (compare Table S3). The first specific term is “systemic acquired resistance” (turquoise label in Figure 6a). This term points to the possible underlying general biological stress response. Moreover, it relates to many other enriched terms related to defense response. CC enrichment of upregulated genes showed the terms “extracellular region” and “plant‐type cell wall” (compare Table S3) and the less than 100 downregulated genes resulted in five significant BP terms: “plant epidermis development,” “root development,” “root system development,” “water transport,” and “fluid transport” (Figure S5). For a full list of enriched terms, see Table S3. L‐Ara feeding upregulated the gene expression level of many genes in ARAK1‐OE seedlings and ARA1 was one of them (red dot; Figure 6b). Based on these results and focused on stress as well as pathogen response, six upregulated genes were tested independently by qPCR for their expression levels in response to L‐Ara. For programmed cell death *LURP1* (late upregulated in response to *Hyalorperonosopora parasitica* 1), *SARD1* (SAR deficient 1) and *RMG1* (resistance methylated gene 1) were chosen. Moreover, for stress responses connected to signaling a peroxidase (*PER52*) and the two transmembrane proteins *CRK13* (cysteine‐rich receptor‐like protein kinase 13) and *CYSTM8* (cysteine‐rich transmembrane module 8) were tested. All gene expression levels were calculated relative to the housekeeping gene *EF1a*, and measured expression levels of the WT were set to 1 (Figure 7). All genes showed a significant upregulation in response to 3 mM L‐Ara when compared with WT, which confirmed the data obtained by DEG: *LURP1* increased 49.8‐fold, *SARD1* about 11.1‐fold, and *RMG1* about 7.8‐fold, *CRK13* about 3.4‐fold, *PER52* about 97.8‐fold, and *CYSTM8* about 8.8‐fold.

In brief, transcriptomic analysis of ARAK1‐OE plants fed with L‐Ara revealed strong upregulation of defense‐ and stress‐related genes, including those linked to SAR and PCD.

## 3 Discussion

Previously, arabinose accumulation was thought to be toxic for plants, leading to lethal phenotypes (Dolezal and Cobbett 1991). However, this hypothesis was challenged when plants with a knock‐out mutation in the *ARA* gene were found to accumulate even higher levels of L‐arabinose (L‐Ara) without exhibiting any visual phenotypes (Behmüller et al. 2016). Arabinokinase, the enzyme encoded by the *ARA* gene, plays a key role in the recycling pathway of L‐Ara as it is responsible for the recycling of free L‐Ara from various sources (metabolism, degradation, L‐Ara uptake by plant roots). Under normal conditions, the function of this recycling pathway does not seem essential for regular plant growth and development. However, when the kinase domain of ARA1 was transiently overexpressed and combined with L‐Ara feeding, severe problems were observed in tobacco leaves (Behmüller et al. 2016). This finding prompted us to generate ARAK1‐OE lines that overexpressed the kinase domain of ARA1 to investigate this process in more detail.

Both ARAK1‐OE lines grew normally on soil or plates without L‐Ara supplementation. However, in the presence of as little as 1 mM L‐Ara, overexpressing seedlings showed significant retardation in root growth and even cell death compared with wildtype seedlings. The overexpression lines also accumulated much higher amounts of UDP‐L‐Ara*p* in response to L‐Ara feeding, suggesting that the intermediate L‐Ara‐1P is rapidly converted to UDP‐L‐Ara. In contrast, feeding L‐Ara to WT seedlings did not cause root damage, even though the level of UDP‐L‐Ara*p* increased about threefold. In ARAK1‐OE lines, this increase in UDP‐L‐Ara*p* was much stronger (about eightfold, compare Figure 4c) and suggested that the activity of the kinase domain of ARA1 regulates the recycling pathway to UDP‐L‐Ara. Moreover, plants developed normally unless a critical threshold of UDP‐L‐Ara was exceeded, at which point severe cellular reactions like cell death occurred, resembling pathogen‐induced hypersensitive responses. Other key UDP‐sugars, such as UDP‐glucose and UDP‐galactose, did not change in concentration (Figure 4a,b) and the amount of arabinose within the cell wall differed only slightly between WT and ARAK1‐OE (compare Figure S4). This indicated that neither the pool of UTP nor the activity of UDP‐sugar pyrophosphorylase (USP) is the bottleneck in L‐Ara recycling. Instead, the availability of L‐Ara‐1P appears to be the limiting factor. These rescue experiments suggested that the toxicity phenotype diminishes as soon as the UDP‐L‐Ara pool is below a critical concentration. We further noticed that direct contact of the root to L‐Ara‐containing medium is necessary to see the described phenotype, suggesting that L‐Ara is not systemically transported, but taken up and metabolized locally.

Next, we searched for variations in the cell walls of ARAK1‐OE seedlings due to L‐Ara feeding by using CoMPP, a powerful tool to identify structural changes in the cell walls of plants (Kračun et al. 2017). In our analyses, we focused on the major hemicelluloses, the polymers that contain L‐Ara. As shown in Figure 5, quantitative differences between WT and ARAK1‐OE for most signals were rather low. However, the abundance of several AGPs was higher in the pectic (CDTA) fraction of WT cell walls when compared with overexpressor lines. Interestingly, signal intensities were even higher in the hemicellulose (NaOH) fraction and the allocation was exactly the opposite: the signal for AGP polymers was highest in ARAK1‐OE. These findings suggest that AGPs of L‐Ara fed ARAK1‐OE are more tightly integrated into the cell wall. This hypothesis is supported by the work of Tan et al. (2013), who identified a specific proteoglycan complex in *Arabidopsis*, named APAP1, in which AGPs are covalently linked to both pectin and arabinoxylan, revealing a structural cross‐linking role for AGPs within the wall matrix. Their findings provided biochemical evidence that AGPs can anchor into both the pectic and hemicellulose fractions, aligning with our observed shift of AGPs to more tightly associated fractions in ARAK1‐OE plants. Moreover, AGPs may interact with hemicelluloses or cellulose through strong hydrogen bonds or ester linkages. This corresponds well with our observed reduction in root cell size of overexpressor seedlings growing on L‐Ara and points to more rigid and thus less extensible cell walls in ARAK1‐OE. Willats and Knox (1996) showed that adding Yariv reagent to *Arabidopsis* cell cultures also strongly reduces cell size by cross‐linking AGPs. This effect was dose‐dependent and showed a reduction of cell size at 30‐μM Yariv and a full stop in cell expansion at 70 μM (Gao and Showalter 1999). Our studies also revealed a tight dose‐dependent phenotype in ARAK1‐OE with an about 50% reduction in the length of root cells at an L‐Ara concentration of as low as 1 mM (Figure 2a). If 10 mM L‐Ara is added, the seedlings will start to germinate but typically arrest and die within a few days (Figure S1). This tight cross‐linking of AGPs by addition of Yariv reagent was described to trigger a PCD by a yet unknown mechanism (Gao and Showalter 1999; Guan and Nothnagel 2004). Moreover, CoMPP analyses showed a higher signal intensity in the hemicellulose fraction of ARAK1‐OE cell walls when β1,3‐glucan callose was tested (Figure 5). The antibody BS‐400‐2 binds to β1,3‐glucan callose and is often a good marker for pathogen‐, wound‐, and stress‐defense (Guan and Nothnagel 2004). This observation supports a mimic of diverse stresses that are also caused by cross‐linked L‐Ara polymers. Changes in AGP presentation, induced by the addition of β‐glucose‐Yariv, is one possibility for such a modification. The overexpression of ARAK1 seems to present AGPs with a slight modification, which is potentially recognized and thus similar to Yariv treatment. Whereas several studies were conducted with Yariv, our study is the first one suggesting modification by biosynthesis due to higher concentration of UDP‐L‐Ara. Although cell death after Yariv treatment is frequently observed, the exact mechanism is yet unknown. Two possible mechanisms are suggested (Guan and Nothnagel 2004; Tang et al. 2006; Maurer et al. 2010). One is that AGP‐Yariv disturbs the regular function of AGPs. Another possibility might be the disruption of AGP plasmamembrane interaction. In recent years many cell wall integrity receptors have been identified (Wolf et al. 2012). Many of them are present in small gene families, which complicates functional analysis. Unfortunately, a method for sequencing of sugar polymers is currently also not available, which would allow to identify the structure of molecules for defense gene and programmed cell death.

To investigate underlying biological processes, we examined potential gene expression responses to L‐Ara feeding by comparing transcriptomic data of ARAK1‐OE and WT seedlings grown on arabinose. A general increase in gene expression levels (Figure 6b) was observed in mutant seedlings in response to feeding. GO enrichment analysis of downregulated genes revealed five general biological process terms: “plant epidermis development,” “root development,” “root system development,” “water transport,” and “fluid transport.” All of them may contribute to the observed phenotype, although no specific pathway was identified. Among the 25 most upregulated BP terms (with more than 500 genes), general terms involved in plant defense response were prominent. Additionally, cellular component enrichment analysis of all upregulated genes highlighted terms like “extracellular region” and “plant‐type cell wall” (see Table S3), aligning with the observed changes in ARAK1‐OE cell walls. The first specific terms identified were “response to salicylic acid” and “systemic acquired resistance” (Figure 6a), suggesting the involvement of genes potentially linked to the cell death responses observed in root cells. To validate the transcriptomic findings, we independently tested six genes upregulated in our datasets (Table S2) using qPCR. These genes, known to be induced during pathogen recognition, programmed cell death (PCD), hypersensitive responses, or other stress‐related events, include *LURP1*, *SARD1*, *RMG1*, *CRK13*, *PER52*, and *CYSTM8* (Figure 7). *LURP1* is the most highly upregulated gene in the LURP cluster in response to pathogen recognition and is essential for basal defense and resistance (Knoth and Eulgem 2008). *SARD1* encodes a transcription factor and key regulator of isochorismate synthase 1 (ICS1), a crucial enzyme in the salicylic acid synthesis pathway essential for immune defenses (Zhang et al. 2010). *RMG1* is strongly induced by flg22, a peptide that activates the flagellin receptor, and is known to play a role in disease resistance (Yu et al. 2013). *CRK13*, a receptor‐like protein kinase, is induced early in response to *Pseudomonas* infection and its overexpression triggers PCD in *Arabidopsis* (Acharya et al. 2007). The peroxidase *PRX52* is involved in oxidative stress response and lignin biosynthesis (Fernández‐Pérez et al. 2015) while *CYSTM8*, a cysteine‐rich transmembrane protein responsive to salicylic acid, contributes to stress tolerance (Mendes et al. 2020). Our qPCR analyses confirmed that all tested genes were indeed upregulated in ARAK1‐OE plants in response to L‐Ara feeding compared with WT. These results confirm the substantial gene induction observed in transcriptomic analyses, consistent with patterns seen after pathogen infection and programmed cell death. Overall, the RNA sequencing analysis supports the phenotypic observation that enhanced UDP‐L‐Ara levels trigger programmed cell death in the roots of ARAK1‐OE lines.

Finally, it is surprising that a rather small change in the metabolite UDP‐L‐Ara causes programmed cell death. This experimental system opens the door to study the role of AGPs in root shaping and PCD.

## 4 Experimental Procedures

### 4.1 ARAK1 Overexpression Lines

For all experiments described in this manuscript, *A. thaliana* ecotype Columbia 8 (Col‐8) was used either as wild type or as the basis for the creation of mutant lines. Arabinokinase1‐kinase‐domain overexpressing lines were created by using the floral dip method described in Davis et al. (2009) and *Agrobacterium tumefaciens* strain GV3101 carrying the vector *pGreen0229‐35Sprom‐ara1(1.8)‐C‐strep*, which was previously prepared in our lab (see Behmüller et al. 2016). Mutant plants were selected by Basta treatment, and ARAK1 overexpression was confirmed by quantitative PCR (see below).

### 4.2 Plant Growth

For plant growth on agar plates, *A. thaliana* seeds were surface sterilized and placed on 0.5× MS‐agar plates (Murashige & Skoog medium including modified vitamins [Duchefa #M0245, Haarlem, The Netherlands]; pH 5.7 and 0.8% plant agar [Duchefa #P1001]) either without added sugar or containing L‐arabinose (L(+)‐arabinose, #101492 Merck, Darmstadt, Germany). The concentration of L‐Ara differs and is described in each experiment. Following stratification (2 days, 4°C) the plates were placed in short day conditions (8 h light at 23°C and 16 h darkness at 18°C) and plants were grown for up to 14 days, unless mentioned otherwise. Seedlings were harvested around noon. Samples were snap frozen in liquid nitrogen and stored at −80°C until use.

### 4.3 Analyses of Roots

For measurements of whole root length, seedlings were grown on 0.5× MS‐agar plates supplemented with either 1 mM or 3 mM L‐Ara or without any sugar added (described above). Root growth was documented daily by photography from day 3 to 14 post germination. Root length was measured using the software ImageJ (Schneider et al. 2012).

Cell length and width of individual root cells of WT and ARAK1‐OE #7 mutant seedlings (14 days old), grown on 0.5× MS‐agar plates supplemented with 1 mM L‐Ara, were measured using light microscopy. Root cells at different positions along the root were measured: (1) directly below the hypocotyl at the beginning of the root, (2) in the middle of the total root length, and (3) at the end directly above the root tip.

For determining dead root cells, seedlings were cultivated on 0.5× MS‐agar plates containing different L‐Ara concentrations (up to 10 mM) for 7 to 14 days, respectively. Seedlings were carefully removed from the plates using forceps and placed into Evans Blue staining solution (0.01% in ddH_2_O) for 5 min, as described in Baker and Mock (1994). After staining, the seedlings were washed twice in ddH_2_O until no further elution of staining solution occurred from the tissue. Seedlings were examined immediately after staining using a Leica MZFLM‐stereo microscope, equipped with a digital camera.

### 4.4 RNA Isolation, cDNA Preparation, and Gene Expression Analyses

For RNA extraction, 20 to 25 mg seedlings (14 days old) were collected from 0.5× MS‐agar plates with 3 mM or without L‐Ara (as described above). Samples were snap‐frozen in liquid nitrogen and stored at −80°C until analysis. Total RNA was extracted with TRI‐Reagent according to the supplier's protocol (Sigma Aldrich, St. Louis, USA), purified via silica spin columns (Thermo Fisher Scientific, Waltham, MA, USA) and eluted in ddH_2_O(DEPC). Any residual genomic DNA was digested using RNase‐free DNase (EN0521, Thermo Fischer Scientific). First‐strand cDNA was synthesized from 2 μg total RNA by M‐MuLV Reverse Transcriptase (RevertAid EP0441, Thermo Fischer Scientific) combined with an anchored oligo(d)T primer‐mix according to the manufacturer's instructions. The obtained cDNA was used as a template for gene expression analysis using quantitative PCR (qPCR).

For gene expression analysis, qPCR was performed on an AriaMx cycler (Agilent Technologies, California, USA) using SYBR Green according to the manufacturer's instructions. The following primers were used: For overexpression of *ARAK1* (At4g16130), we used ARAK1_fwd: 5′‐GTATCTTCATCTGCTGCTGTG‐3′ and ARAK1_rev: 5′‐GCTCCAACAATGTGATTCTCC‐3′; for other gene expression analyses we used *LURP1* (At2g14560, LURP1_fwd: 5′‐TGATAACGAGTGCGGACGGTAAG‐3′, LURP1_rev: 5′‐TGCATGGTCATCATCTTCCCTCTC‐3′), *SARD1* (At1g73805, SARD1_fwd: 5′‐AAGTGTGACGGTGAGAAACGG‐3′, SARD1_rev: 5′‐CACAAACCACAACGCCTTGAC‐3′), *RMG1* (At4g11170, RMG1_fwd: 5′‐GCGTGGAAAGGGTAAGGAAGAG‐3′, RMG1_rev: 5′‐CATGCAAGCCATCATACCCGAC‐3′), *CRK13* (At4g23210, CRK13_fwd: 5′‐TCTTGTCGCGAAACTTCAGCATAG‐3′, CRK13_rev: 5′‐CCTTGCTTTGTAGGGTCAAAGAGG‐3′), *PRX52* (At5g05340, PRX52_fwd: 5′‐TCAGCGGTTGAGAAAGCATGTCC‐3′, PRX52_rev: 5′‐ATTCCAGTTCGGTCCTCCAAGG‐3′), and *CYSTM8* (At3g22235, CYSTM8_fwd: 5′‐TTCATCAGGGCCGTACACAAGTC‐3′, CYSTM8_rev: 5′‐ATGGCAGCACAACATCCTTCGG‐3′); *AtEF1a* was used as a housekeeping gene (At5g60390, primers: EF1a‐qPCR‐fwd: 5′‐GACCAACTCTTCTTGAGGCTCTTGAC‐3′, EF1a‐qPCR‐rev: 5′‐GGCACCGTTCCAATACCACCAATC‐3′). Means of resulting *C*_t_ values were used for calculation of gene expression relative to *AtEF1a* according to (Pfaffl et al. 2002). Gene expression levels of WT seedlings were set to 1 and fold changes in ARAK1‐OE#7 seedlings compared with WT were calculated (*n* = 3).

### 4.5 UDP‐Sugar Analyses

UDP‐sugars were isolated from 10‐ to 25 mg seedlings using a liquid–liquid extraction with subsequent solid phase extraction according to (Behmüller et al. 2014). Samples were reconstituted in 50 μL mobile phase A (20.0 mmol/L TEA‐AA adjusted to pH 6.0) and analyzed by UHPLC‐MS. For measurements, a Vanquish Flex UHPLC system was coupled by a HESI‐II probe to a Q Exactive mass spectrometer, both from Thermo Fisher Scientific. Samples (10‐ μL injection) were separated on a YMC‐Triart C18 column (150 × 2.0 mm i.d.; dP: 1.9 μm; pore size: 12 nm, column temperature 22°C). The mobile phase B was ACN; for the separation, a linear gradient from 0.0% to 1.5% B in 17.0 min followed by a second linear gradient from 1.5% to 90% B at a flow rate of 200 μL/min was used. Mass spectrometry of nucleotide sugars was conducted in targeted selected ion monitoring using negative ionization mode at −3.50 kV and a resolution setting of 70,000 at 200 *m/z*. Data generation was performed with Chromeleon 7.2.10; peak integration was performed with the included Cobra Wizard tool. Nucleotide sugar standards were purchased from Complex Carbohydrate Research Center (University of Georgia, Athens, GA, USA). UDP‐L‐Ara*f* was prepared from UDP‐L‐Ara*p* by an enzyme assay using reversibly glycosylated polypeptide 1 (RGP1; Rautengarten et al. 2011) and used to determine the position of UDP‐L‐Ara*f* on the chromatogram, but not for quantification. As no purified standards are available for UDP‐L‐Ara*f*, we calculated its available amount (in %) relative to the whole pool of pyranose and furanose.

### 4.6 Cell Wall Sugar Analysis

For cell wall sugars analysis, we adapted the method described in Reboul et al. (2011): Seedlings (10–20 mg) were snap frozen in liquid nitrogen and homogenized using a ball mill (Model PM200; Retsch, Düsseldorf, Germany) 2 × 1 min at 30 Hz. Following the addition of 0.5 mL EtOH (70%), samples were vortexed, centrifuged (10 min, 16,200 × g), and the supernatant was carefully removed. This extraction was repeated once with 0.5 mL EtOH (70%), twice with 0.5 mL methanol/chloroform 1:1 (v/v), and finally with 1 mL acetone. Samples were dried using a vacuum centrifuge at 30°C and hydrolyzed in 250 μL 2 M TFA by autoclaving (60 min, 121°C). After hydrolysis the dried samples (vacuum centrifuge, 45°C) were resuspended in 750‐μL ddH_2_O. Prior to measurement, samples were diluted 1:1 in ddH_2_O and analyzed using HPEAC‐PAD (Model ICS3000; Dionex, Sunnyvale, CA, USA). Separation of monosaccharides was achieved on a CarboPac PA20 column (3 × 150 mm) using a flow rate of 0.45/min.

### 4.7 Glycan Microarray Analysis of Alcohol‐Insoluble Residues (AIR) and Comprehensive Microarray Polymer Profiling (CoMPP)

Roots of wild type and ARAK1‐OE seedlings grown on 0.5× MS‐agar plates supplemented with 3 mM L‐Ara for 2 weeks were collected and homogenized. Cell walls (alcohol‐insoluble residues, AIR) were extracted in 75% ethanol until the supernatant was transparent. Cell wall polysaccharides and glycoproteins were sequentially extracted from AIR in 50 mmol/L CDTA (solubilizing pectins, glycoproteins) and 4 mol/L NaOH (solubilizing hemicelluloses, glycoproteins). Glycan microarray analysis (Moller et al. 2007) was performed as described in Kračun et al. (2017). Arrays were printed as distinct dots onto nitrocellulose membranes with an ArrayJet Sprint microarray printer (ArrayJet, Roslin, UK) and probed with ~40 cell wall probes, including antibodies and carbohydrate binding modules (Table S1). For controls, primary antibodies were heat‐inactivated prior to use. Seedling growth, cell wall extraction, and probing were performed in triplicates.

### 4.8 Genome‐Wide Transcriptional Analyses

Total RNA was extracted from *A. thaliana* seedlings as described above. RNA sequencing and library preparation was performed by Novogene GmbH (München, Germany). Libraries were prepared using the TruSeq RNA Sample Prep Kit v2 (Illumina, Berlin, Germany), and library quantification was performed with Qubit 2.0 (Invitrogen, Waltham, MA, USA). All libraries were sequenced on a HiSeq 2500 Illumina platform in the paired‐end mode with a length of 150 nucleotides. The achieved sequencing depth was 30 Mbp per sample. Demultiplexed FASTA files were supplied for further RNA sequencing analysis. Reads were pseudo‐aligned using kallisto v0.44.0 (Bray et al. 2016) with TAIR10 representative isoforms and cDNA data obtained from phytozome12 (Goodstein et al. 2012). The results were concatenated in R (v3.6.3). Mean, standard deviation, and error calculations were performed with base v3.6.3 and tidyverse v1.3.1. Log2‐foldchange was calculated by log2((mean_tpmOX + 1)/(mean_tpmWT + 1)). DEG analysis was performed with edgeR v3.28.1 (Robinson et al. 2010) from counts and normalized with the calcNormFactors function. Genes with a false discovery rate (FDR) lower than or equal to 0.01 are considered differentially expressed. The RNA‐seq experiment was performed twice, and resulting data were averaged for WT (Col‐8) as well as overexpressor lines (ARAK1‐OE #7). A mothertable (Table S2) was collated from DEG analysis including tpm (transcripts per million), count data, and gene annotations from TAIR10 (Berardini et al. 2015) as well as Araport11 (Cheng et al. 2017) and Mercator (using MapMan bins; Lohse et al. 2014). Functional enrichment analyses were performed using the GO (Gene Ontology) term annotation (obtained from www.arabidopsis.org on the 31.01.2023) with topGO v2.38.1 (topGO: Enrichment Analysis for Gene Ontology. R package version 2.54.0; https://rdrr.io/bioc/topGO/). A full list of GO enrichment terms can be found in Table S3.

## 5 Statistics

Statistical tests (unpaired *t* tests) and graphical representation related to gene expression and cell wall sugar analyses as well as root cell lengths were performed using GraphPad Prism 9 (version 9.1.2). Analyses of functional enrichment analyses were calculated from the classic Fisher exact test, and multiple hypothesis testing adjustment was calculated with Benjamini–Yekutieli correction (Benjamini and Yekutieli 2001).

## Author Contributions

EIK, MCH, and RT conceptualized the manuscript; MCH and RT wrote the main parts of the manuscript; EIK, MCH, MH, WH, CR, and KH performed experiments, evaluated data, and contributed to the corresponding methodological sections.

## Conflicts of Interest

The authors declare no conflicts of interest.

## Accession Numbers

ARABINOKINASE1 (ARA1) ‐ **AT4G16130**

ARABINOKINASE2 (ARA2) ‐ **AT3G42850**

ELONGATION FACTOR 1‐ALPHA (AtEF1a) ‐ **At5g60390**

LATE UPREGULATED IN RESPONSE TO HYALOPERONOSPORA PARASITICA (*LURP1*) ‐ **At2g14560**

SYSTEMIC ACQUIRED RESISTANCE DEFICIENT 1 (*SARD1*) ‐ **At1g73805**

RESISTANCE METHYLATED GENE 1 (*RMG1*) ‐ **At4g11170**

CYSTEINE‐RICH RLK (RECEPTOR‐LIKE PROTEIN KINASE) 13 (*CRK13*) ‐ **At4g23210**

PEROXIDASE 52 (*PRX52*) ‐ **At5g05340**

CYSTEINE‐RICH TRANSMEMBRANE MODULE 8 (*CYSTM8*) – **At3g22235**

## Supporting information


**Figure S1.** **Growth of *Arabidopsis thaliana* wild type (WT) and arabinokinase kinase domain overexpressing lines (ARAK1‐OE #7 and #25) on 10 mM L‐Ara.** Seedlings were grown on 0.5x MS‐agar plates supplemented with 10 mM L‐Ara for (a) 7 days and (b) 12 days (bar = 1 cm).

**Figure S2.** **Root tips of 14 days old *A*** ***. thaliana*** **seedlings stained with Evans Blue.** Root tips of WT (a), ARAK1‐OE #7 (b), and #25 (c) grown on 5 mM L‐Ara; on 7.5 mM L‐Ara: WT (d), ARAK1‐OE #7 (e), and #25 (f) and on 10 mM L‐Ara: WT (g), ARAK1‐OE #7 (h), and #25 (i) for 14 days and stained with Evans Blue. Arrows indicate dead cells (appearing blue due to Evans Blue staining). Asterisks mark areas of sprouting root hair (bar = 100 μm).

**Figure S3.** **Root tips of 7 days old *A. thaliana* seedlings stained with Evans Blue.** Root tips of seedlings grown on 0.5x MS‐agar plates for 7 days without L‐Ara supplemented ((a) WT, (b) ARAK1‐OE #7, and (c) #25) or supplemented with 3 mM L‐Ara ((d) WT, (e) ARAK1‐OE #7, and (f) ARAK1‐OE #25). Arrows indicate dead cells (appearing blue due to Evans Blue staining). Asterisks mark areas of sprouting root hair (bars = 100 μm in (a‐d) and 200 μm in (e, f).

**Figure S4.** **Cell wall monosaccharide composition in *A. thaliana* wild type (WT) and overexpressing lines (ARAK1‐OE #7, #25) with 3 mM or without L‐Ara feeding.** 14 days old seedlings grown on 0.5x MS‐agar plates containing (a) no sugar or (b) 3 mM L‐Ara were used to determine their cell wall sugar composition. After TFA hydrolysis, free sugars were measured using HPLC (pulsed amperometric detection). The following sugars are shown: fucose (Fuc), rhamnose (Rha), arabinose (Ara), galactose (Gal), xylose (Xyl), mannose (Man), galacturonic acid (GalA), glucuronic acid (GlucA); statistical differences were evaluated using ANOVA (Dunett's test, *p* < 0.05, *n* = 4 replicates per line, mean ± SD).

**Figure S5** **GO plot from GO enrichment of DEG.** The plot displays the outcome of Gene Ontology (GO) enrichment analyses conducted separately for significantly downregulated (on the left) and upregulated (on the right) genes from the RNA sequencing experiment comparing ARAK1‐OE and WT cultivated on 3 mM L‐Ara. Each plot showcases the top enriched terms ranked from top to bottom. Since, depending on the specificity of the terms, more or fewer genes are assigned to them, the percentage of significant genes in the respective terms is shown as a percentage (effect size). The total number of significant genes in the respective term, on the other hand, is represented by dot size. Thus, specific terms appear smaller but further right on the x‐axis. Among upregulated genes, the terms “response to salicylic acid” and “systemic acquired resistance” stand out as specific. Among the downregulated genes, the enrichment of “root development”, “root system development” and “plant epidermis development” is evident. Additionally, the significance of enriched GO terms, determined through Fisher's exact test with Benjamini–Yekutieli correction, is represented by a green color scale. A full list of results from GO enrichment can be found in Table S3.

**Table S1** **Data of CoMPP analyses.** Table shows raw data of comprehensive microarray polymer profiling (CoMPP) from *A. thaliana* wild type and ARAK1‐OE #7 seedlings grown on 3 mM L‐arabinose as well as corresponding antibody legends and summarizing heatmap. Cell wall components were extracted from AIR samples with diaminocyclohexane‐tetraacetic acid (CDTA) and NaOH. Data consist of 3 technical replicates per each of the two biological replicates.

**Table S2** **Mothertable.** Results of differential gene expression (DEG) analysis of the RNA sequencing experiment and corresponding functional annotations are shown. ARAK1‐OE and WT grown on 0.5x MS‐agar plates supplemented with 3 mM L‐Ara are compared. Column A contains gene identifiers of *A. thaliana* (locus ID). Genes used for qPCR are highlighted in column B. Transcript abundances resulting from Kallisto mapping normalized to tpm (transcripts per million) are shown in columns C‐F. The color gradient in columns C‐F intensifies with higher abundance in an orange to red color scale. ARAK1‐OE samples are displayed in columns C‐D and WT samples in columns E‐F. The log2FC, p‐value, and p‐adjust are shown in columns G, H, and I. Coloring of log2FC: upregulated genes are shown in red, downregulated genes in blue; both with an increasing color gradient. p‐adjust coloring ranges from intense green (significant, <0.001) to white (non‐significant, >0.05). Columns J and K contain symbols and names of listed genes. Columns L and M contain the annotation in binary categories from MapMan. Columns O‐V show annotation information from TAIR10 (www.arabidopsis.org) and Araport11 (www.araport.org).

**Table S3** **GO enrichment.** The results from the GO enrichment analysis performed on significant up‐ and downregulated genes from DEG analysis independently for the three categories biological process (BP), molecular function (MF), and cellular component (CC) are represented in independent data sheets in the spreadsheet.

## Data Availability

The data that support the findings of this study are available from the corresponding author upon reasonable request.
