# Supplementary figures and images for: Arabinokinase Limits the Flux of Arabinose Into Nucleotide Sugars to Prevent Toxicity
Source: Plant Direct. 2025 Jul 22;9(7):e70094. doi: 10.1002/pld3.70094 (PMC12281597; doi:10.1002/pld3.70094)

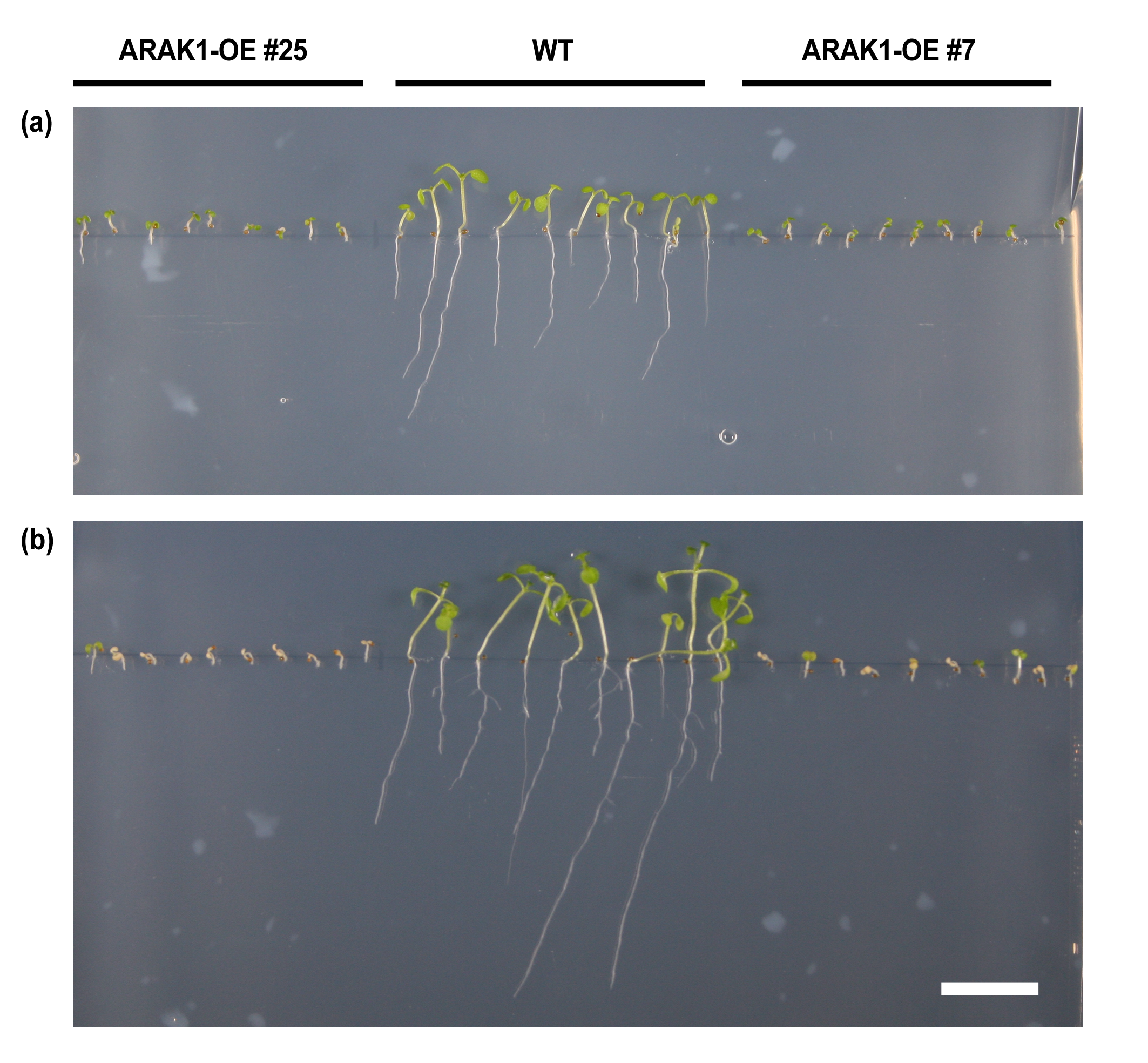

Supplement: Supplementary file 1 — Figure S1. Growth of Arabidopsis thaliana wild type (WT) and arabinokinase kinase domain overexpressing lines (ARAK1‐OE #7 and #25) on 10 mM L‐Ara. Seedlings were grown on 0.5x MS‐agar plates supplemented with 10 mM L‐Ara for (a) 7 days and (b) 12 days (bar = 1 cm). [file PLD3-9-e70094-s006.tif]

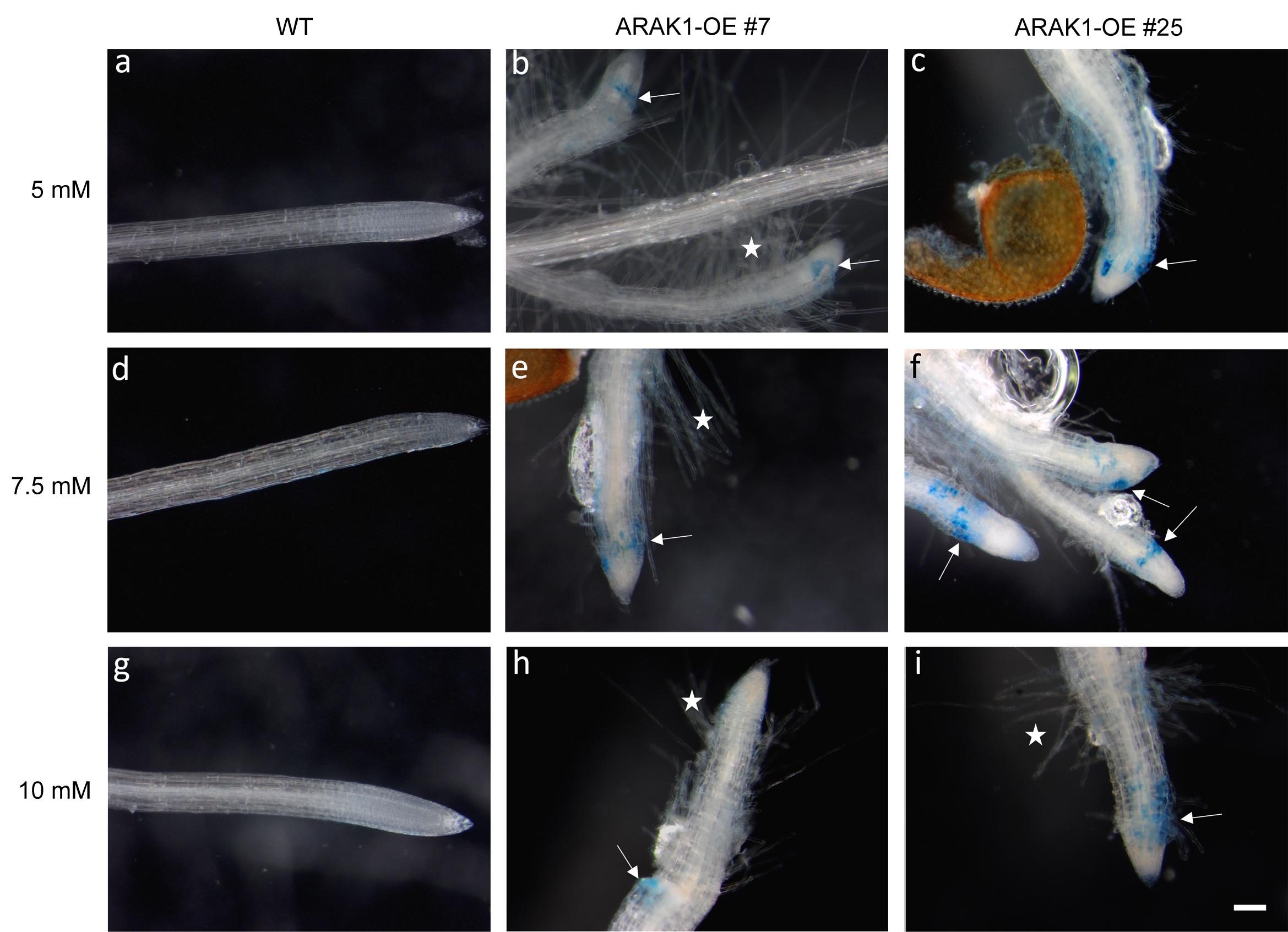

Supplement: Supplementary file 2 — Figure S2. Root tips of 14 days old A . thaliana seedlings stained with Evans Blue. Root tips of WT (a), ARAK1‐OE #7 (b), and #25 (c) grown on 5 mM L‐Ara; on 7.5 mM L‐Ara: WT (d), ARAK1‐OE #7 (e), and #25 (f) and on 10 mM L‐Ara: WT (g), ARAK1‐OE #7 (h), and #25 (i) for 14 days and stained with Evans Blue. Arrows indicate dead cells (appearing blue due to Evans Blue staining). Asterisks mark areas of sprouting root hair (bar = 100 μm). [file PLD3-9-e70094-s004.tif]

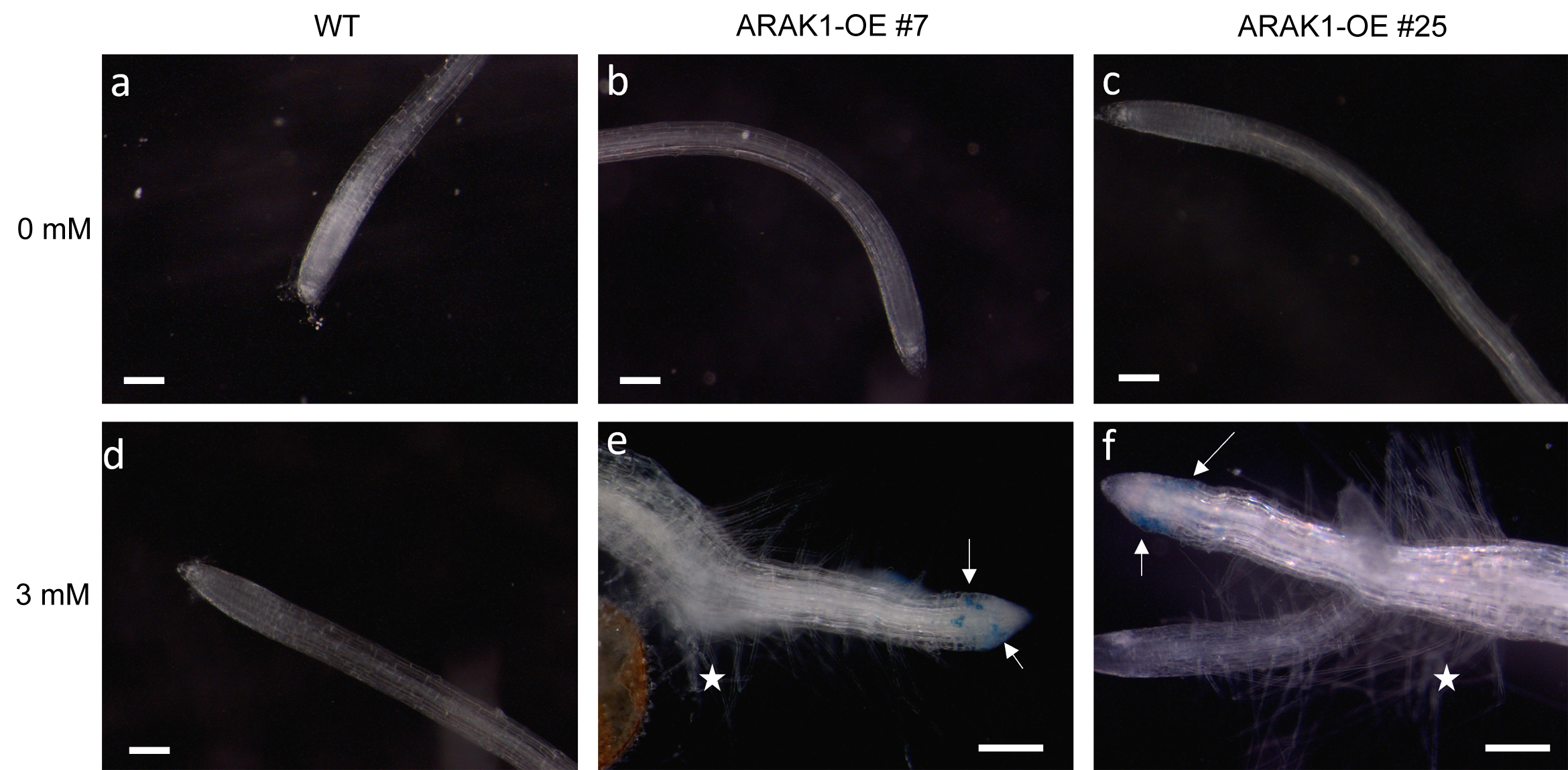

Supplement: Supplementary file 3 — Figure S3. Root tips of 7 days old A. thaliana seedlings stained with Evans Blue. Root tips of seedlings grown on 0.5x MS‐agar plates for 7 days without L‐Ara supplemented ((a) WT, (b) ARAK1‐OE #7, and (c) #25) or supplemented with 3 mM L‐Ara ((d) WT, (e) ARAK1‐OE #7, and (f) ARAK1‐OE #25). Arrows indicate dead cells (appearing blue due to Evans Blue staining). Asterisks mark areas of sprouting root hair (bars = 100 μm in (a‐d) and 200 μm in (e, f). [file PLD3-9-e70094-s007.tif]

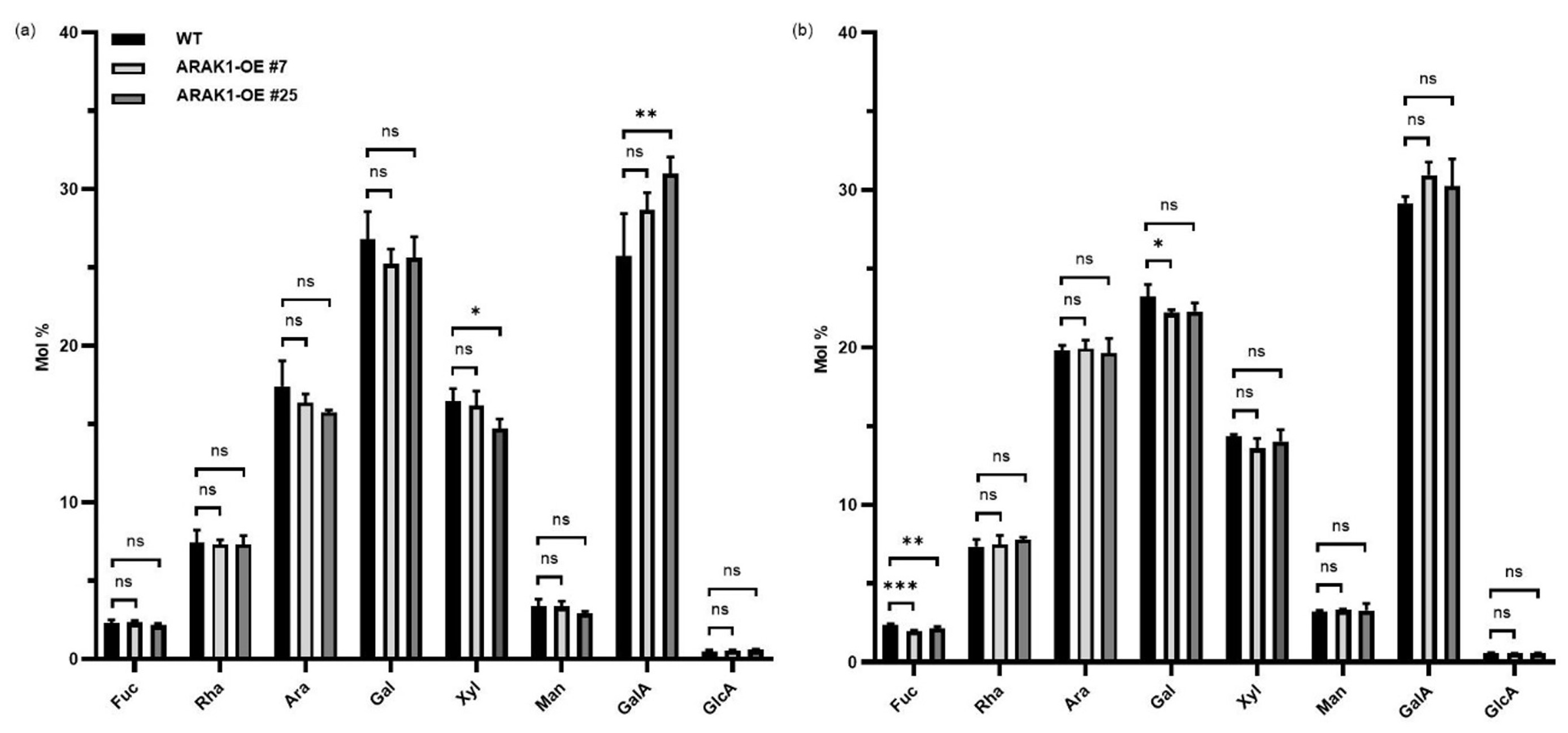

Supplement: Supplementary file 4 — Figure S4. Cell wall monosaccharide composition in A. thaliana wild type (WT) and overexpressing lines (ARAK1‐OE #7, #25) with 3 mM or without L‐Ara feeding. 14 days old seedlings grown on 0.5x MS‐agar plates containing (a) no sugar or (b) 3 mM L‐Ara were used to determine their cell wall sugar composition. After TFA hydrolysis, free sugars were measured using HPLC (pulsed amperometric detection). The following sugars are shown: fucose (Fuc), rhamnose (Rha), arabinose (Ara), galactose (Gal), xylose (Xyl), mannose (Man), galacturonic acid (GalA), glucuronic acid (GlucA); statistical differences were evaluated using ANOVA (Dunett's test, p < 0.05, n = 4 replicates per line, mean ± SD). [file PLD3-9-e70094-s002.jpg]

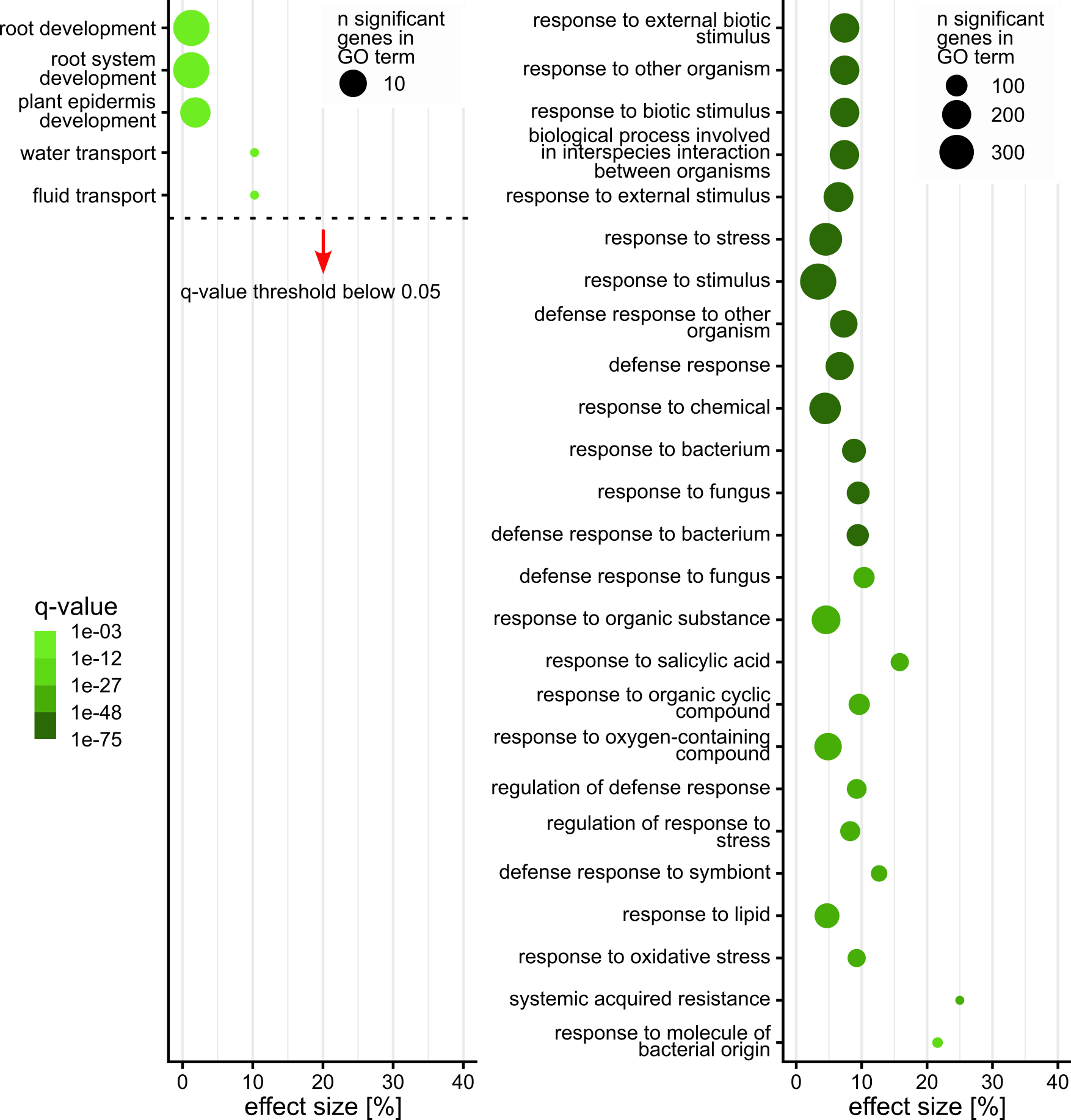

Supplement: Supplementary file 5 — Figure S5 GO plot from GO enrichment of DEG. The plot displays the outcome of Gene Ontology (GO) enrichment analyses conducted separately for significantly downregulated (on the left) and upregulated (on the right) genes from the RNA sequencing experiment comparing ARAK1‐OE and WT cultivated on 3 mM L‐Ara. Each plot showcases the top enriched terms ranked from top to bottom. Since, depending on the specificity of the terms, more or fewer genes are assigned to them, the percentage of significant genes in the respective terms is shown as a percentage (effect size). The total number of significant genes in the respective term, on the other hand, is represented by dot size. Thus, specific terms appear smaller but further right on the x‐axis. Among upregulated genes, the terms “response to salicylic acid” and “systemic acquired resistance” stand out as specific. Among the downregulated genes, the enrichment of “root development”, “root system development” and “plant epidermis development” is evident. Additionally, the significance of enriched GO terms, determined through Fisher's exact test with Benjamini–Yekutieli correction, is represented by a green color scale. A full list of results from GO enrichment can be found in Table S3. [file PLD3-9-e70094-s005.tif]
